# Further clarification of cognitive processes of prospective memory in schizophrenia by comparing eye-tracking and ecologically-valid measurements

**DOI:** 10.1038/s41537-024-00465-1

**Published:** 2024-04-05

**Authors:** Hang Li, Qi Wang, Wen-Peng Hou, Dong-Yang Chen, Yu-Shen Ding, Zhi-Fang Zhang, Wei-Wei Hou, Sha Sha, Ning-Bo Yang, Qi-Jing Bo, Ya Wang, Fu-Chun Zhou, Chuan-Yue Wang

**Affiliations:** 1 The National Clinical Research Center for Mental Disorders & Beijing Key Laboratory of Mental Disorders Beijing Anding Hospital & the Advanced Innovation Center for Human Brain Protection, Capital Medical University, School of Mental Health, Beijing, China; 2 Beijing Fengtai Mental Health Center, Beijing, China; 3 Vanke School of Public Health, Tsinghua University, Beijing, China; 4 First Affiliated Hospital of Henan University of Science and Technology, Luoyang, China; 5 School of Psychology, Capital Normal University, 23A Baiduizi, Haidian District, Beijing, 100073 China

**Keywords:** Schizophrenia, Human behaviour

## Abstract

The aim of this study is to compare ecologically-valid measure (the Cambridge Prospective Memory Test, CAMPROMPT) and laboratory measure (eye-tracking paradigm) in assessing prospective memory (PM) in individuals with schizophrenia spectrum disorders (SSDs). In addition, eye-tracking indices are used to examine the relationship between PM and other cognitive domains in SSDs patients. Initially, the study sample was formed by 32 SSDs patients and 32 healthy control subjects (HCs) who were matched in sociodemographic profile and the performance on CAMPROMPT. An eye-tracking paradigm was employed to examine the differences in PM accuracy and key cognitive processes (e.g., cue monitoring) between the two groups. Additional 31 patients were then recruited to investigate the relationship between PM cue monitoring, other cognitive functions, and the severity of clinical symptoms within the SSDs group. The monitoring of PM cue was reflected in total fixation time and total fixation counts for distractor words. Cognitive functions were assessed using the Chinese version of the MATRICS Consensus Cognitive Battery (MCCB). The Positive and Negative Syndrome Scale (PANSS) was applied to assess psychopathology. SSDs patients exhibited fewer total fixation counts for distractor words and lower PM accuracy compared to HCs, even though they were priori matched on CAMPROMPT. Correlation analysis within the SSDs group (63 cases) indicated a negative correlation between PM accuracy and PANSS total score, and a positive correlation with working memory and attention/vigilance. Regression analysis within the SSDs group revealed that higher visual learning and lower PANSS total scores independently predicted more total fixation counts on distractor words. Impairment in cue monitoring is a critical factor in the PM deficits in SSDs. The eye-tracking laboratory paradigm has advantages over the ecologically-valid measurement in identifying the failure of cue detection, making it a more sensitive tool for PM deficits in patients with SSDs.

## Introduction

Schizophrenia is a debilitating mental disorder characterized by cognitive impairments, including deficits in memory^1,2^. Prospective memory (PM), the cognitive ability to remember future tasks or intentions, exhibits complexity due to the delay between intention formation and action execution^3^. It is critical to daily living, with 50−80% of the impact of memory impairment on everyday life attributed to deficits in PM^4^. Patients with PM impairments may forget to take medication or exhibit poor adherence to other treatments, which can negatively impact social function and quality of life, as well as increase the risks of relapse, hospitalization, or even suicide^5–7^. Therefore, addressing impaired PM is crucial for patients’ recovery and improved clinical management.

PM is commonly categorized into event-based prospective memory (EBPM) and time-based prospective memory (TBPM). EBPM refers to remembering to execute future intentions when external cues/events occur, while TBPM involves proactively carrying out future intentions after a time interval^8^. The PM processing consists of the following stages: intention encoding, intention retention, intention initiation, and intention execution^9^. The intention initiation phase includes two components that are impaired in schizophrenia: cue detection, which refers to the recognition of cues for future intentions, and intention retrieval, which involves retrieving intentions from long-term memory after recognizing PM cues^10^.

Whether the cue is non-focal or focal is a significant factor influencing PM processing^11,12^. A focal PM task is characterized by some overlap between the definition of PM cues and the processing of ongoing tasks. In contrast, there is no overlap between the definition of PM cues and the processing of ongoing tasks in a non-focal PM task^13^. According to the multiprocessing theory^14^, strategic monitoring is the key factor in intention retrieval in non-focal PM, which is closely linked to the interaction between the anterior prefrontal cortex (aPFC) and the dorsal frontoparietal network (superior frontal lobule, superior parietal lobule, and precuneus)^10^. These findings indicate that in non-focal PM, there is a more prominent involvement of top-down processes^12^.

Measurements of PM share several common components. They include the encoding of an intention to be performed in the future, an ongoing task during the delay period, and a PM cue to signal it is time to execute the intention^15^. Researchers have developed a range of measurements to assess PM. Among these are laboratory measurements, ecologically valid (or eco-valid) measurements, and self-report measurements^3^. Laboratory measurements often use a dual-task paradigm, a method that has been extensively employed in the literature. This paradigm entails an embedded PM task within an ongoing task, succeeded by a period of delay. It is believed that laboratory measurements offer greater control and manipulation of specific variables to address specific theoretical questions^14^. However, it has been considered that the dual-task laboratory paradigms have low ecological validity, as participants perform only one type of PM task repeatedly^16^. One way to address this limitation is by improving the laboratory paradigm. Recently, certain studies have employed eye-tracking paradigm to explore PM^17–20^. Eye-tracking studies on PM commonly utilize a visual search task involving the presentation of multiple stimuli, offering a more profound understanding of PM processing^21,22^. For instance, the total number of fixations on distractors (stimuli other than PM cues and targets in a visual search task) accurately portrayed the individual’s sensitivity in strategic monitoring of PM cues^22,23^.

In neuropsychology, the concept of ecological validity is utilized to denote the extent to which the results obtained from a cognitive assessment are able to accurately predict actual behaviors exhibited in the real world^24^. Eco-valid cognitive measurements refer to assessments or tests that aim to capture cognitive processes and abilities in a way that closely resembles real-world or everyday situations. There are currently four standardized eco-valid measurements of PM^10^, including the Cambridge Prospective Memory Test (CAMPROMPT)^25^, Virtual Week^26^, the Memory for Intentions Test (MIST) ^27^, and the Royal Prince Alfred Memory Test^28^. These tests have also shown good psychometric properties^29^. Of these, the CAMPROMPT is widely utilized clinical assessment tool for PM and has been reported to be sensitive to the impacts of neuropsychiatric and neurodegenerative disorders^30–33^. While eco-valid PM measures are likely to reflect actual real-world behavior, they are not without their problems. There appears to be no consensus on the definition in the literature, nor any established means of classification for evaluating or determining a study’s ecological validity. Researchers rarely clarify how they have assessed a study’s ecological validity^34^. Different researchers have even used different definitions and interpretations^35,36^.

As a higher-order complex cognitive functions, PM relies on a complex network circuitry that involves the anterior prefrontal cortex (aPFC), the dorsal frontoparietal network, the ventral frontoparietal network, and their interconnections^10,37–39^. Differences of PM performance may occur due to disparities in type of cue, type of task, the nature of ongoing task, and individual diffierences^29^. Therefore, ecologically valid measurements may struggle to capture the full complexity of PM and make it challenging to isolate specific cognitive processes, potentially oversimplifying or omitting important elements that could impact PM functioning. Furthermore, eco-valid measurements often lack experimental control, making it difficult to establish causal relationships between cognitive processes and outcomes.

A recent meta-analysis has demonstrated that eco-valid PM measurements are associated with more severe impairment of PM (indicated by PM summary/composite scores) compared to dual-task laboratory tests in individuals with schizophrenia^3^. For EBPM, authors found a similar result using fixed-effect model, but the mixed-effect analysis did not reveal any differences between eco-valid and laboratory measures. The pooled standard mean deviation was 1.2 in 8 studies using eco-valid measures and 1.0 in 19 studies using dual-task laboratory measures (between-group Q-value = 0.8, *P* = 0.384). It should be cautious when interpreting these results due to a high degree of heterogeneity in study population and measurements of PM. However, in our review of the literature, we have not come across any prior studies that have utilized both measurement tools in a single study. Therefore, it would be worthwhile to compare these two PM assessment tools in the same sample. Additionally, employing an eye-tracking paradigm during laboratory tests could better simulate real-world situations where participants are required to identify PM cues among multiple stimuli^17,21,22^.

The aim of the present study was to compare an ecologically-valid measure (CAMPROMPT) and a laboratory measure (a non-focal eye-tracking EBPM paradigm) in assessing PM in patients with SSDs. In addition, eye-tracking indices were used to investigate the relationship between PM and other cognitive domains in SSDs patients. The current study’s hypotheses comprised the following: ①Despite the seemingly intact EBPM performance assessed by the CAMPROMPT, the eye-tracking paradigms can still capture impaired monitoring of PM cues in SSDs patients, a critical component of PM deficits in SSDs. ②PM accuracy and cue monitoring ability indicated by eye-tracking indices are associated with certain cognitive domains in MCCB in SSDs.

## Materials and methods

### Participants

The study was conducted in Beijing Anding hospital. Initially, 32 SSDs patients (iSSDs) and 32 HCs matched in sociodemographic profile and the performance on CAMPROMPT were invited to participate in the study to compare the eco-valid and eye-tracking based laboratory PM assessment. Additional 31 patients were then recruited to investigate the relationship between PM cue monitoring, other cognitive functions, and the severity of clinical symptoms within the SSDs group. Consequently, 63 SSDs (tSSDs) from the outpatients and inpatients departments were recruited in the study.

Criteria for inclusion were as follows: age range of 18 to 50 years; a minimum of 9 years of education, the medication plan had not been altered in the last 3 months, and participants were clinically stable; IQ＞80, measured by the short version of Wechsler Adult Intelligence Scale-Revised in China (WAIS—RC)^40^; right-handed; all patients fulfilled the diagnostic criteria for schizophrenia and other psychotic disorders (e.g., schizoaffective disorders) as outlined in the Fifth edition of the Diagnostic and Statistical Manual of Mental Disorders (DSM-5)^41^. A research psychiatrist confirmed the diagnosis using the MINI International Neuropsychiatric Interview (MINI 7.0.2)^42^.

Exclusion criteria were as follows: patients with severe neurological diseases (history of craniocerebral trauma or infection, brain tumor, cerebrovascular disease, epilepsy, etc.) or other severe medical conditions; patients who received electroconvulsive therapy or neuromodulation within the past 6 months; pregnant or lactating women.

The healthy controls (HCs) were matched for gender, age, and years of education with the patient group. Additionally, MINI screening was performed to rule out the presence of any diagnosable mental disorders.

No prior study has compared the laboratory PM accuracy between SSDs and HCs when matching their eco-valid PM measures. Nonetheless, in a recent study using the same eye-tracking PM paradigm (without matching eco-valid measures), a very large effect size has been reported to detect a significant difference in PM accuracy between SSDs and HCs^19^. We conservatively expected an effect size of Cohen’s *d* = 0.8 for the present study. The following formula was used to determine the appropriate sample size for: *n* = (Z_α/2_ + Z_β_)2 *2/(Cohen’s d)2. Z_α/2_ is the critical value at α/2 (for a confidence level of 95%, α is 0.05 and the critical value is 1.96), Z_β_ is the critical value at β (for a power of 80%, β is 0.2 and the critical value is 0.84). This study aimed to recruit 60 participants (30 in SSDs group and 30 in HCs group) assuming approximately 10% missing data due to unusable eye-tracking data and dropouts.

The institutional review board of Beijing Anding Hospital provided approval for this study. All participants provided written informed consent before entering the study.

### PM assessments

PM was assessed using the Chinese version of the Cambridge Prospective Memory Test (C-CAMPROMPT). This assessment tool has demonstrated its utility across a range of clinical conditions, including chronic and first episode schizophrenia, showing good ecological validity^32,33^. During this test, participants are instructed to complete three EBPM tasks and three TBPM tasks at varying intervals. These tasks are performed while concurrently engaging in an ongoing activity following both verbal and written instructions.

**Fig. 1:**
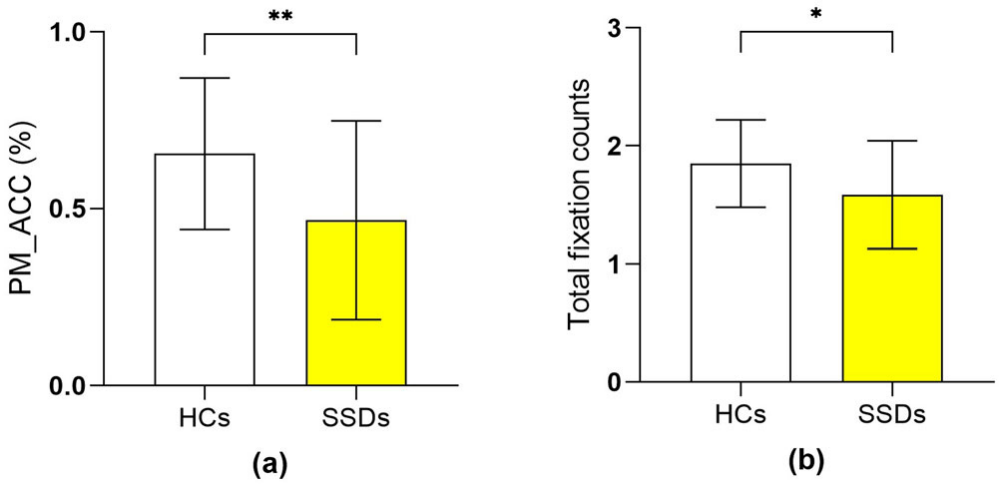
Behavioral and eye tracking measures of PM in individuals with iSSDs and HCs. SSDs=Patients with schizophrenia spectrum disorders, PM prospective memory, HCs Healthy controls, PM_ACC the accuracy of PM trials, Total fixation counts=total fixation counts for distractor words. **a** The comparison of PM_ACC between the iSSDs and HCs. **b** The comparison of the fixation counts for distractor words between iSSDs and HCs.

Detailed description of the eye-tracking paradigm and apparatus used in this study can be found in previous literature^19^. Briefly, it is a typical non-focal dual-task paradigm. Initially, a simple line drawing appeared in the center of the screen. Following this, four distinct words were presented on the screen. Participants were then tasked with to determine whether one of the words corresponded to the object in the previously displayed picture. Participants were directed to press the “J” key if any word matched the preceding picture and press the “F” key if none of the words matched (the ongoing task). Participants were asked to press the “spacebar” whenever an animal word (e.g., elephant) appeared (the PM task). There were 2 blocks of PM tasks in the PM session. Within each block, 6 PM trials were spread across 74 ongoing trials. Half of the 80 trials included the target word. A one-minute break was planned between the two blocks. Figure 1 illustrates the sequence of the experiment.

### Cognition, clinical assessments and procedures

The MATRICS Consensus Cognitive Battery (MCCB) was administered to assess the cognitive functions of all participants^43^. In the Chinese version of the MCCB, there are 7 cognitive domains, consisting of processing speed, working memory, verbal learning, attention/vigilance, visual learning, reasoning and problem-solving, and social cognition^44^.

PANSS was used to measure the psychopathology in patients with schizophrenia^45^. The PANSS was conducted by experienced psychiatrists who had received training to ensure the reliability and accuracy of the results.

All the assessments were conducted in the morning. The IQ test was administered first, followed by the eye-tracking PM paradigm, C-CAMPROMPT, and MCCB. The PANSS was rated on the same days as the above tests.

### Statistical analysis

Behavioral data analysis includes the evaluation of response time and accuracy for both ongoing task trials (OT_RT, OT_ACC) and PM trials (PM_RT, PM_ACC). Eye-tracking data extraction utilized Data Viewer 3.2. Blink artifacts and fixations (gaze) below the 80 ms threshold were removed. Analysis of eye movement data was performed using regions of interest (ROI), each with a resolution of 238 × 144 pixels and a visual angle of about 5° × 3°. The analysis included the following eye movement indices: ①total fixation counts for distractor words, indicating the overall number of gazes on distractor words in ongoing trials; ②total fixation time for distractor words, representing the accumulated duration of all fixations on the distractors during ongoing trials; ③time from first fixation to response, indicating the duration between the first gaze within the ROI of PM cues and the response. ④time to first fixation, denoting the interval between the onset of word stimuli and the initial fixation within the region of interest of PM cues; The total fixation time for distractors and total fixation counts for distractors within these indices were utilized to signify the process of PM cue monitoring. The time from first fixation to responses indicates the time spent on intention retrieval and execution, while the time to first fixation offers insight into an individual’s alertness to stimuli.

Statistical analyses were conducted using the SPSS 24.0 software package. The comparison of continuous variables across different groups employed the T-test or Mann−Whitney U-test. The chi-square test was used for group comparisons involving categorical variables. To explore the relationships between PM cue monitoring, PM_ACC, MCCB scores, and PANSS total score in the SSDs group, Spearman’s rank correlation or Pearson correlation was applied as appropriate. All tests were two-tailed, and the significance level was set at *P* < 0.05.

ANCOVAs were conducted to investigate whether the difference in PM_ACC between iSSDs and HCs could be solely attributed to changes in the variance of cue monitoring and basic neurocognitive functions, as measured by the MCCB.

Stepwise multiple linear regression analyses were used to identify factors that were independently associated with performance on PM cue monitoring in the SSDs group. In the regression analyses, The total fixation counts for distractors was designated as the dependent variable, and all variables that displayed significant correlations with PM cue monitoring were entered as independent variables.

## Results

Table 1 presents the basic demographic variables in the iSSDs (*n* = 32) and HCs, along with the clinical characteristics for iSSDs. Age, gender, year of education, and IQ were matched between the two groups. There was no significant difference in the performance of the EBPM on C-COMPROMPT.

**Table 1:** Demographic and clinical characteristics of individuals with iSSDs and HCs.

	iSSDs (*n* = 32)		HCs (*n* = 32)		Statistics	df	*P* value
	*N*	Percent	*N*	Percent	X²		
Men	14	43.75	12	37.50	0.259	1	0.611
on ACM	9	28.1	-	-	-	-	-
	Mean	SD	Mean	SD	*t*	df	*P* value
Age (years)	31.97	8.00	30.59	7.05	0.730	62	0.468
IQ	105.31	9.82	109.39	10.66	−0.683	62	0.497
Education (years)	14.75	4.06	15.38	3.20	−1.594	62	0.116
Course of illness (years)	6.46	6.67	-	-	-	-	-
PANSS-positive	12.22	5.43	-	-	-	-	-
PANSS-negative	14.53	4.82	-	-	-	-	-
PANSS-general	25.91	7.63	-	-	-	-	-
PANSS-total	52.66	15.52	-	-	-	-	-
Medication (CPZ eq mg/day)	388.01	259.20	-	-	-	-	-

*SSDs* Patients with schizophrenia spectrum disorders, *HCs* Healthy controls, *ACM* anticholinergic medication, *IQ* intelligence quotient, *PANSS* Positive and Negative Syndrome Scale, *PANSS-positive* Positive subscale score of the PANSS, *PANSS-negative* Negative subscale score of the PANSS, *PANSS-general* General psychopathology subscale score of the PANSS, *PANSS-total* Total score of the PANSS, *CPZ eq* Chlorpromazine equivalence, *SD* Standard deviation.

Among the thirty-two patients, one was prescribed a first-generation antipsychotic medication (fluphenazine), one received a combination of first- and second- generation antipsychotics (fluphenazine and aripiprazole), while the remaining thirty cases exclusively took second-generation antipsychotic medications. Nine patients were administered with anticholinergic medications (benzhexol).

To examine the association between use of anticholinergic medications and PM performance, comparative analyses were performed between patients who concurrently took anticholinergic medications and those who did not, in terms of PM_ACC, total fixation counts for distractor words, and total fixation duration for distractor words. Independent sample *t* test indicated no significant differences between the two groups of patients in PM_ACC (*t* = −0.825, *P* = 0.416), total fixation counts for distractor words (*t* = 1.387, *P* = 0.176), and total fixation duration for distractor words (*t* = 0.892, *P* = 0.380).

Table 2 presents the average performance of the ongoing and PM tasks, eye movement indices and MCCB scores in iSSDs and HCs. ISSDs patients performed significantly lower performance in PM_ACC, total fixation counts for distractor words, working memory, speed of processing, visual learning, and verbal learning compared to HCs. Figure 1 illustrates the differences in PM-ACC and total fixation counts for distractor words between the iSSDs group and HCs. Even after controlling for MCCB total scores through ANCOVA, the PM_ACC of the iSSDs patients remained significantly lower than that of the HCs (F _(1,58)_ = 4.19, *P* = 0.045). However, after further controlling for both MCCB total scores and total fixation counts for distractor words by ANCOVA, the disparity in PM_ACC between the two groups was no longer present (F _(1,58)_ = 1.077, *P* = 0.304).

**Table 2:** Comparison of cognitive function, eye-tracking data, PM performance, and ongoing task performance between individuals with iSSDs and HCs.

	iSSDs (*n* = 32)		HCs (*n* = 32)		*t*	df	*P* value
	M	SD	M	SD			
Speed of processing	45.28	9.17	53.78	7.91	−3.971	62	<0.001
Attention/vigilance	50.25	9.17	54.13	8.75	−1.73	62	0.089
Working memory	45.22	9.54	53.50	9.30	−3.515	62	<0.001
Verbal learning	45.75	10.74	52.69	9.96	−2.679	62	0.009
Visual learning	45.34	13.31	54.28	7.85	−3.272	50	0.002
Reasoning and problem-solving	50.06	12.55	53.53	8.57	−1.291	55	0.202
Social cognition	48.81	10.87	53.31	6.72	−1.992	52	0.052
Overall composite	45.69	11.27	55.38	7.50	−4.048	54	<0.001
PM_ACC (%)	0.47	0.28	0.65	0.21	−3.005	62	0.004
PM_RT (ms)	2078.18	543.72	2119.24	405.81	−0.342	62	0.733
OT_ACC (%)	0.94	0.05	0.97	0.02	−3.507	38	0.001
OT_RT (ms)	2205.32	512.01	2287.4	396.69	−0.717	62	0.476
Total fixation time (ms)	361.57	113.09	390.95	86.94	−1.165	62	0.248
Total fixation counts	1.59	0.46	1.85	0.37	−2.536	62	0.014
Time to first fixation (ms)	703.53	185.57	677.04	158.94	0.613	62	0.542
First fixation to response (ms)	1392.08	477.40	1439.02	347.91	−0.449	62	0.655

Total fixation counts total fixation counts for distractor words, Total fixation time total fixation duration for distractor words, Time to first fixation the interval between the onset of word stimuli and the first fixation within the ROI of PM cues, First fixation to response the duration between the first gaze within the ROI of PM cues and the response.

*SSDs* Patients with schizophrenia spectrum disorders, *PM* Prospective memory, *HCs* Healthy controls, *PM_ACC* the accuracy of PM trials, *PM_RT* the response time of PM trials, *OT_ACC* the accuracy of ongoing trials, *OT_RT* the response time of ongoing trials, *SD* standard deviation.

Correlation analysis in SSDs patients (*n* = 63) revealed that PM_ACC was significantly and positively correlated with working memory (*r* = 0.27, *P* = 0.031) and attention/vigilance (*r* = 0.398, *P* = 0.01). Conversely, there was a significant negative correlation between PM_ACC and PANSS total score (*r* = −0.326, *P* = 0.009). Additionally, the total fixation counts for distractor words in the SSD patients (*n* = 63) exhibited a significant positive correlation with visual learning (*r* = 0.29, *P* = 0.008) and a significant negative correlation with PANSS total score (*r* = −0.283, *P* = 0.01)(see Supplementary Table 1).

Furthermore, to explore the independent contribution of various cognitive domain functions to cue monitoring in the SSDs group, a regression analysis was performed. The total fixation counts for distractor words was entered as the dependent variable, while the correlated cognitive domain scores as the independent variables. Stepwise multiple linear regression analysis revealed that higher visual learning (*β* = 0.254, *P* = 0.04) and lower PANSS total scores (*β* = −0.243, *P* = 0.049) contributed to better strategic monitoring (R² = 0.143) (see Supplementary Table 2).

## Discussion

This study was the first to compare two types of PM assessment tools (ecological validity measure vs. laboratory measure) in SSDs patient. The two hypotheses were both confirmed. Despite the seemingly comparable PM performance between the two groups as assessed by the C-CAMPROMPT, SSDs patients still exhibited lower PM_ACC and total fixation counts for distractor words in the eye-tracking paradigm compared to HCs. This indicates the limitations of ecological validity measure, which cannot precisely examine the processing of PM. Furthermore, PM accuracy was correlated with working memory and attention/vigilance, and cue monitoring ability was predicted by higher visual learning on MCCB.

These findings were not quite in line with the results of the previous meta-analysis, which generally favored eco-valid measures^3^. In this meta-analysis, 8 studies with eco-valid and 19 studies with dual-task laboratory tests were included in the subgroup analyses for EBPM. As we re-examined the included studies, we found the cue focality may be a significant confounding factor. One the one hand, most of the previous dual-task tests employed a focal paradigm. For instance, in the EBPM session of Wang et al. 2008’s study, participants were instructed to press a pre-specified key when they saw an animal character in a four-character words presented on the screen, while simultaneously engaged in an ongoing task (judging if the word is a Chinese idiom). On the other hand, an example of an EBPM task for CAMPROMPT is to give a book to the tester when he/she comes across a question consisting of the phrase ‘Mount Tai’, which is a non-focal PM task. As we mentioned earlier, non-focal tasks are more mentally demanding and consume more cognitive resources. This would be more challenging for patients with SSDs, whose cognitive resources are already limited. Factors beyond cognitive abilities, such as environmental influences or individual differences, can also confound the results of eco-valid measurements, making them less likely to yield reliable and consistent results. For example, CAMPROMPT allows participants to use various strategies to help them remember and conduct the tasks. As it turns out, some participants have used the assistant strategies while others have not. On the contrary, laboratory-based tests typically have more controlled conditions, allowing for precise measurement of specific cognitive processes.

After controlling for MCCB scores in this study, PM_ACC in SSDs patients remained lower than that in HCs. This suggests that PM impairment in SSDs patients is independent of other cognitive functions, consistent with previous research findings^46–48^. Nevertheless, when both MCCB scores and the total fixation counts for distractor words were included as covariates, the difference in PM_ACC between the two groups disappeared, verifying that cue monitoring ability is a key cognitive component for PM^20^. In non-focal PM tasks, monitoring PM cues relies more on strategic monitoring, which includes top-down attention and memory processes used for monitoring PM cues in the environment and maintaining the intention^12,37^. A functional magnetic resonance imaging study demonstrated that patients with schizophrenia exhibited reduced activation in multiple brain regions, including the prefrontal cortex, anterior cingulate cortex (ACC), parietal and temporal cortices, as well as subcortical areas such as the parahippocampal gyrus and caudate when performing a PM task compared to healthy controls. This suggested that the deficits in PM observed in patients with schizophrenia may be attributed to impairments in attentional control and allocation, leading to failures in PM cues monitoring^49^.

The total fixation counts for distractors (stimuli other than target and cue words) in visual search tasks can effectively assess participants’ strategic monitoring abilities. This study found that visual learning can independently predict strategic monitoring abilities. This is an intriguing finding, as the eye-tracking technology used in this study is based on visual search tasks. One of the primary functions the brain must accomplish when acquiring novel visual information is the recognition of the incoming material. Brain areas implicated in the process of recognition encompass the inferior temporal cortex, the cerebellum and the superior parietal cortex. In the context of recognition tasks, there is a notable increased activation in the left inferior temporal cortex and a decrease in activation within the right superior parietal cortex. The process of recognition is significantly facilitated by neural plasticity-the capacity of the brain to reconfigure itself in response to fresh information^50,51^.

This is the first study to compare clinical measure (C-CAMPROMPT) and laboratory measure (eye-tracking paradigm) in assessing PM in SSDs patients. This comparison has provided us a more profound understanding of the prevalent cognitive processing deficits in these patients. Nevertheless, caution is warranted in interpreting the results due to potential limitations. Firstly, the sample size was relatively small in this study, which could constrain the applicability of the findings, despite the matched psychopathology and sociodemographic variables between groups. Secondly, the design of the cross-sectional study inhibited the investigation of causality in the relationships between PM_ACC and cue monitoring. Lastly, other commonly used eco-valid PM tests, such as Virtual Week, were not investigated.

In summary, this study further clarified the cognitive processes of PM in patients with SSDs by comparing laboratory and ecologically-valid measurements. The eye-tracking laboratory paradigm has advantages over the ecologically-valid measurement in identifying the failure of cue detection, making it a more sensitive tool for PM deficits in patients with SSDs. In the future, a combination of eye-tracking paradigm and eco-valid tools should be used to comprehensively assess participants’ PM functions. Furthermore, PM cue monitoring was verified to be a critical component in the process of PM, making it a potential target for interventions for PM impairments in SSDs.

### Supplementary information

Supplementary information

## Data Availability

The data that support the findings of this study are available on request from the corresponding author.
