# Supplementary material for: Further clarification of cognitive processes of prospective memory in schizophrenia by comparing eye-tracking and ecologically-valid measurements
Source: Schizophrenia (Heidelb). 2024 Apr 5;10(1):41. doi: 10.1038/s41537-024-00465-1 (PMC10997606; doi:10.1038/s41537-024-00465-1)
Supplement: Supplementary file 1 — Supplementary information [file 41537_2024_465_MOESM1_ESM.docx]

**Supplementary Table 1**

Correlation between the accuracy of PM trials and the eye movement indices and MCCB scores in SSDs (n=63).

|  | PM_ACC | Total fixation counts | SOP | A/V | WM | VISL | VERL | RPS | SC |
| --- | --- | --- | --- | --- | --- | --- | --- | --- | --- |
| PM_ACC |  |  |  |  |  |  |  |  |  |
| Total fixation counts | 0.511^**^ |  |  |  |  |  |  |  |  |
| Speed of processing | 0.195 | 0.108 |  |  |  |  |  |  |  |
| Attention/vigilance | 0.398^**^ | 0.246 | 0.463^**^ |  |  |  |  |  |  |
| Working memory | 0.273^*^ | 0.208 | 0.304^*^ | 0.186 |  |  |  |  |  |
| Visual learning | 0.199 | 0.293^*^ | 0.403^**^ | 0.378^**^ | 0.392^**^ |  |  |  |  |
| Verbal learning | 0.104 | 0.130 | 0.542^**^ | 0.522^**^ | 0.307^*^ | 0.552^**^ |  |  |  |
| Reasoning and problem-solving | 0.211 | 0.022 | 0.465^**^ | 0.205 | 0.399^**^ | 0.202 | 0.234 |  |  |
| Social cognition | 0.227 | 0.229 | 0.260^*^ | 0.314^*^ | 0.082 | 0.239 | 0.153 | 0.117 |  |
| PANSS-total | -0.326^**^ | -0.283^*^ | -0.091 | -0.309^*^ | -0.197 | -0.158 | -0.125 | -0.227 | -0.160 |

*PM* prospective memory, *MCCB* the MATRICS Consensus Cognitive Battery, *SSDs* schizophrenia spectrum disorders, *Total fixation counts* total fixation counts for distractor words, *PM_ACC* the accuracy of PM trials, *SOP* Speed of processing, *A/V* Attention/vigilance, *WM* Working memory, *VISL* Visual learning, *VERL* Verbal learning, *RPS* Reasoning and problem-solving, *SC* Social cognition, *PANSS-total* total score of the PANSS, *p＜0.05, **p＜0.01.

**Supplementary Table 2**

Results of stepwise multiple regression analysis in SSDs (n=63).

| Measure | Predictor | Beta | p-value | 95%CI |  |
| --- | --- | --- | --- | --- | --- |
| Total fixation counts | Visual learning | 0.254 | 0.04 | 0.001, 0.021 | |
| R²=0.143 | PANSS-total | -0.243 | 0.049 | -0.014, -0.00029 | |
| F_（2,62）_=5.013, p=0.01 | |  |  |  |  |

*SSDs* schizophrenia spectrum disorders, *Total fixation counts* total fixation counts for distractor words, *PANSS-total* total score of the PANSS.
